# Association of Human Leukocyte Antigen DRB1*15 and DRB1*15:*01* Polymorphisms with Response to Immunosuppressive Therapy in Patients with Aplastic Anemia: A Meta-Analysis

**DOI:** 10.1371/journal.pone.0162382

**Published:** 2016-09-09

**Authors:** Shan Liu, Qing Li, Ying Zhang, Qiushuang Li, Baodong Ye, Dijiong Wu, Li Wu, Hanti Lu, Conghua Ji

**Affiliations:** 1 Center of Clinical Evaluation and Analysis, Zhejiang Provincial Hospital of Traditional Chinese Medicine, Hangzhou, Zhejiang, China; 2 Department of Hematology, Zhejiang Provincial Hospital of Traditional Chinese Medicine, Hangzhou, Zhejiang, China; Children's National Health System, UNITED STATES

## Abstract

This study aimed to review and quantitatively analyze (1) the association of aplastic anemia (AA) with human leukocyte antigen (HLA)-DRB1*15 and HLA-DRB1*15:*01* polymorphisms and (2) the association of HLA-DRB1*15 and HLA-DRB1*15:*01* polymorphisms with response to immunosuppressive therapy (IST) in AA. Published studies have reported conflicting and heterogeneous results regarding the association of HLA-DRB1*15 and HLA-DRB1*15:*01* polymorphisms with response to IST in AA. The PubMed, Embase, Cochrane Library, China National Knowledge Infrastructure, Chinese BioMedical Literature, Wangfang and Chinese Social Sciences Citation Index databases were searched. All relevant publications were searched through December 2015. Odds ratio (*OR*), risk ratio (*RR*), and 95% confidence intervals (*CI*) for the comparison between case–control or cohort studies were evaluated. Finally, 24 articles were identified. For HLA-DRB1*15 and HLA-DRB1*15:*01*, the *OR* (95% *CI*) was 2.24(1.33–3.77), *P* < 0.01 and 2.50(1.73–3.62), *P* < 0.01, respectively; and the overall pooled *RR* was 1.72 (1.30–2.29), *P* < 0.01 and 1.59 (1.29–1.96), *P* < 0.01, respectively. Statistical evidence showed no publication bias (*P* > 0.05). Sensitivity analyses revealed that the results were statistically robust. The meta-analysis suggested that HLA-DRB1*15 and HLA-DRB1*15:*01* polymorphisms might be associated with increased AA risk in Asians. IST might be more effective in HLA-DRB1*15^*+*^ and HLA-DRB1*15:*01*^*+*^ Asian patients with AA than in HLA-DRB1*15− and HLA-DRB1*15:*01−* Asian patients with AA. Future studies with adequate methodological quality on gene–gene and gene–environment interactions and gene treatment may yield valid results.

## Introduction

Aplastic anemia (AA) is a rare, life-threatening hematopoietic stem cell disorder characterized by peripheral blood cytopenia and bone marrow hypoplasia. The prevalence of AA seems to vary in different regions of the world with an annual incidence of two cases per million in Western countries, but a little higher estimated four to seven cases per million in East Asia [1].

A large amount of laboratory and clinical data suggest that immune-mediated destruction of hematopoiesis by activated cytotoxic T cells plays an important role in the pathogenesis of AA. The mechanism of activation of cytotoxic T cells is uncertain, but several potential factors related to antigen recognition, susceptibility of immune response, and secretion of cytokines might be involved. Certain human leukocyte antigen (HLA) alleles were suggested to play a role in the activation of autoreactive T-cell clones in patients with AA [2]. Till date, potential roles of *HLA-DRB1* polymorphisms have been postulated in many types of autoimmune diseases (e.g., systemic lupus erythematosus and lupus nephritis[3], rheumatoid arthritis[4]). As with most autoimmune diseases, AA is genetically associated with alleles of the HLA [5–19].

Conflicting reports exist regarding the correlation of HLA-DRB1*15 and HLA-DRB1*15:*01* polymorphisms with AA. Dhaliwai et al. (2011) demonstrated a significant association of HLA-DRB1*15 polymorphisms with AA, and the odds ratio (*OR*) was 11.09 [9]. However, Sun et al. (2004) reported that HLA-DRB1*15 polymorphisms had no significant association with AA [16]. Unfortunately, no report about AA risk exists in genome-wide association studies. The present study was perhaps the first meta-analysis discussing the relationship of HLA-DRB1*15 and HLA-DRB1*15:*01* polymorphisms with response to immunosuppressive therapy (IST) in AA.

Patients with AA have been treated with IST and hematopoietic stem cell transplantation [1, 2]. However, a lack of HLA-matched sibling donors and the cost of transplantation result in most patients with AA tending to accept IST. IST contains cyclosporin (CsA), antithymocyte globulin (ATG), and antilymphocyte globulin (ALG) [1, 2, 20]. In addition, androgen and traditional Chinese medicine (TCM) also showed some effect on AA [21, 22]. A number of conflicting studies have reported different responses to IST in AA with HLA-DRB1*15 and HLA-DRB1*15:*01* or without HLA-DRB1*15 and HLA-DRB1*15:*01* polymorphisms [14–15, 17–18, 23–31]. When the source of hematopoietic stem cell transplantation is limited, it would be very helpful to predict which patients would benefit from IST.

As many conflicting reports were relatively small in sample size, this study increased the statistical power and evaluated evidences from various studies by summarizing them quantitatively using a meta-analytic approach, to obtain a reliable conclusion. The present study aimed to examine (1) the relationship between *HLA-DRB1* polymorphisms and AA and (2) the association of HLA-DRB1*15 and HLA-DRB1*15:*01* polymorphisms with response to IST in AA.

## Material and Methods

This study was performed following the Quality of Reporting of Meta-analyses guidelines [32] and the recommendations of the Cochrane Collaboration [33]. A protocol for this systematic review was published in PROSPERO with the registration number CRD42015032293(S1 File).

### Search strategy

This study was performed according to the standards of the Preferred Reporting Items for Systematic Reviews and Meta-analyses (PRISMA) criteria [34]. Several databases (PubMed, Embase, Cochrane Library, China National Knowledge Infrastructure, Chinese BioMedical Literature, Wangfang, and Chinese Social Sciences Citation Index databases) were searched through December 2015 for all reports on the association between HLA polymorphism and AA. The search terms were as follows: (‘‘aplastic anemia”) and (‘‘HLA” or ‘‘human leukocyte antigen” or “*DRB1*” or ‘‘major histocompatibility complex” or ‘‘MHC”) and (“cyclosporine” or “antilymphocyte serum” or “immunosuppression” or “antirejection therapy” or “antithymocyte globulin”) (S2 File). No language limitations were used in the search. In addition, references of retrieved reports were also searched, and the study authors were contacted by e-mail to identify additional studies and provide missing data.

### Inclusion and exclusion criteria

The inclusion criteria were as follows: (1) case–control or cohort study, (2) studies concerned with the associations of HLA-DRB1*15 and *HLA*-DRB1*15:*01* polymorphisms with AA or the association of HLA-DRB1*15 and *HLA*-DRB1*15:*01* polymorphisms with response to IST in AA, (3) sufficient data on complete response (CR) and partial response (PR) stratified by HLA-DRB1*15 and *HLA*-DRB1*15:*01* status, and (4) studies providing sufficient data for estimating *OR* or a risk ratio (*RR*) with a 95% confidence interval (*CI*).

The exclusion criteria were as follows: (1) reviews, comments, editorials, basic science, or animal studies; (2) studies that did not reveal genotype frequency, or in which the relevant data could not be obtained after contacting with the authors, and (3) duplicate studies.

### Study selection

The titles and abstracts were examined by two reviewers authors (S Liu and DJ Wu) independently to select eligible studies. Full-text reports of potentially relevant studies were retrieved. When data were overlapped or even duplicated, only the most recent data were included. Full texts were independently examined to decide which articles met the inclusion criteria. Discrepancies in study selection were resolved by a third reviewer (CH Ji).

### Data extraction

Data extraction was conducted by two investigators (S Liu and Q Li) independently using a predetermined extraction form. The third participant (CH Ji) was consulted for discussion to reach an agreement concerning discrepancies. The following items were extracted from each article: first author’s last name, publication year, country, number of cases and controls in case–control studies or number of cases in cohort studies, gene-detection method, genes involved, frequency of *HLA-DRB1* alleles, sex (M/F), age, treatment method, does, follow-up time, response criteria, and Newcastle–Ottawa Scale (NOS).

The outcome was *OR* in case–control studies and *RR* in cohort studies. *OR* was defined as how strongly the presence of HLA-DRB1*15 and HLA-DRB1*15:*01* alleles in patients with AA was associated) with the presence of HLA-DRB1*15 and HLA-DRB1*15:*01* alleles in controls. *RR* was used to show the ratio of probability of the objective response rate (*ORR*) in patients with HLA-DRB1*15 and HLA-DRB1*15:*01* alleles to the probability of the *ORR* in patients without HLA-DRB1*15 and HLA-DRB1*15:*01* alleles. The *ORR* was defined as the sum of CR and PR.

### Quality assessment for individual studies

The study used a scoring system based on the NOS to determine the quality of each article [35]. The NOS ranged between zero (worst) and nine stars (best). Disagreements were settled as described in the preceding section.

### Statistical analysis

All statistical analyses were performed using Stata 12.0 (Stata Corporation, TX, USA). Dichotomous data were reported as *OR* or *RR* (calculated using the chi-square test). The pooled *OR* or *RR* together with the 95% *CI* used for assessing the strength of association was determined using the *Z* test. Heterogeneity across studies was checked using the Cochran’s *Q* statistic and the *I*^2^ test [36]. When *P* value greater than 0.10 for the *Q* test served as no statistical evidence for heterogeneity, the fixed-effects model was used (shown as “M-H”) [37]; otherwise, random-effects model was used (shown as “D+L”) [38]. Subgroup analyses were performed by regions or drugs. To evaluate the stability of outcomes, a sensitivity analysis was performed by sequential omission of individual studies. Harbord’s test was conducted to evaluate the publication bias, with *P* less than 0.05 considered statistically significant [39]. When studying the association of HLA-DRB1*15 or HLA-DRB1*15:*01* polymorphisms with AA, meta-regression was used to reveal whether age, region or NOS score could lead to heterogeneity.

## Results

### Study characteristics

**Fig 1:**
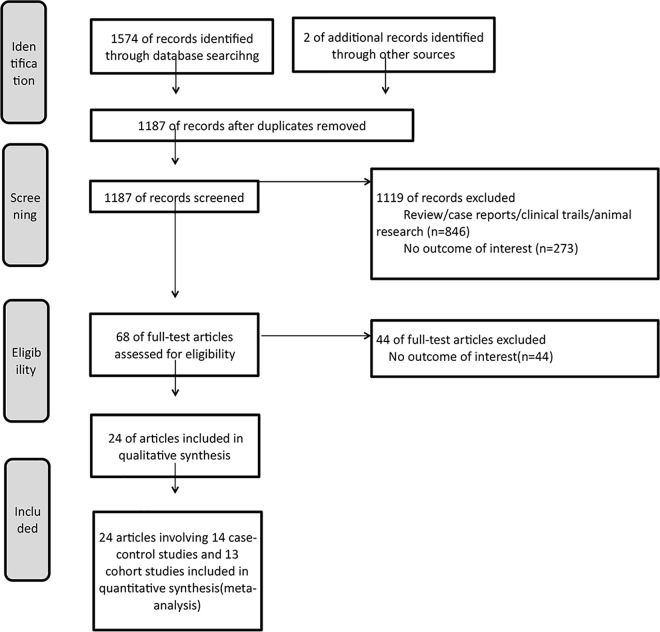
Flow diagram of the study selection process.

The present study met the PRISMA statement requirements (Fig 1 and S1 Table). A total of 1576 published studies were found examining the relationship between HLA polymorphisms and AA. A total of 68 articles were deemed relevant through reading titles and abstracts. Of these, 44 articles were excluded after reading the full text. Finally, 24 articles involving 14 case–control studies and 13 cohort studies were included. Fourteen case–control studies consisted of 938 cases and 5992 controls. Thirteen cohort studies consisted of 609 AA. Tables 1 and 2 list the included studies and their main characteristics. The area of these studies included Asia countries (Japan, Korea, China, Turkey, Malaysia, and Pakistan), Mexico, and Russia. The average score of NOS was 5.6 and 5.3 in case–control and cohort studies, respectively, which revealed that the methodological quality was of average level.

**Table 1:** Characteristics of studies included in the meta-analysis (case–control).

No.	Authors	Year	Country	Numbers		Sex (M/F)		Age		Controls	Detection methods	NOS	Genes
				Cases	Controls	Cases	Controls	Cases	Controls				
1	Song	2008	Korea	109	800	51/58	769/31	22 (1–80)	23 (18–50)	Healthy	PCR-SSP	6	*15:01
2	Sugimori^a^	2007	Japan	140	491	65/75	–	60 (12–92)	–	Healthy	PCR-SSP	6	*15:01
3	Huo	2011	China	115	2264	70/45	–	–	–	Healthy	PCR-SSP	5	*15
4	Liang	2007	China	82	400	56/26	–	2–39	–	Healthy	PCR-SSP	6	*15:01
**5**	Wang	2014	China	43	200	24/19	101/99	40 (18–52)	34 (16–60)	Healthy	PCR-SSP	6	*15:01
6	Yang^a^	2002	China	45	24	28/17	–	22 (8–55)	–	Healthy	PCR-SSP	5	*15:01
**7**	Sun	2004	China	59	30	30/29	16/14	31 (10–58)	30 (15–60)	Healthy	PCR-SSP	6	*15
**8**	Dhaliwal	2011	Malaysia	33	109	20/13	–	18 (13–75)	–	BM donor and Healthy	PCR-SSP	5	*15:01
**9**	Rehman	2009	Pakistan	61	200	39/22	111/89	17 (1–48)	–	Healthy	PCR-SSP	6	*15
**10**	Wang	2014	China	96	600	56/40	–	19 (6–53)	–	BM donor	PCR-SSP	6	*15:01
**11**	Fernandez-Torres	2012	Mexico	36	201	23/13	105/96	11.7 (0.5–63)	–	National Donor	PCR-SSP	6	*15
**12**	Huang^a^	2007	China	40	107	26/16	–	9 (2–14)	–	Healthy	PCR-SSP	5	*15
**13**	Kapustin	2001	Russia	44	100	27/17	–	21 (4–50)	–	Healthy	PCR-SSO	5	*15:01
**14**	Yari	2008	Iran	35	466		–	19 (5–55)		Healthy	PCR-SSP	5	*15

BM,bone marrow;PCR-SSO, polymerase chain reaction with sequence-specific oligonucleotide; PCR-SSP, polymerase chain reaction with sequence-specific primer.

^a^ It is included in the cohort study analysis.

**Table 2:** Characteristics of studies included in the meta-analysis (cohort study).

No.	Authors	Year	Country	AA Numbers	Sex(M/F)	Age	Treatment method	Does	Follow-up time(M)	Detection methods	Response criteria	NOS	Genes
1	Song	2010	Korea	37	19/18	35(3–66)	CsA+ATG/ALG	-	6	PCR-SSP	Champlin	6	*15:01
2	Sugimori	2007	Japan	77	–	–	CsA+ATG	CsA: 6mg/kg/d,1y; then 150–250 ng/ml, >6 m; ATG:15mg/kg/d, 5d	6	PCR-SSP	Camitta	6	*15:01
3	Yang	2002	China	26	-	22(8–55)	CsA+androgen	-	3	PCR-SSP	Zhang	5	*15:01
**4**	Huang	2007	China	40	24/16	9(2–14)	CsA/CsA + ATG +MP	-	6	PCR-SSP	Camitta	5	*15
**5**	Qiao	2010	China	40	22/18	36 (11–79)	CsA	CsA: 5mg/kg/d; then 2.5–3 mg/kg/d	6	PCR-SSP	Zhang	6	*15:01
**6**	Tang	2002	China	29	9/20	24 (12–55)	CsA+androgen+TCM	CsA:6mg/kg/d,10 d;then 3mg/kg/d, >3 m	3	PCR-SSP	–	5	*15:01
**7**	Yang	2004	China	50	36/14	32(13–45)	CsA+androgen	CsA:5mg/kg/d,3 m; then 2.5 mg/kg/d, 3 m	6	PCR-SSO/SSP	Zhang	6	*15:01
**8**	Nakao	1996	Japan	111	55/56	56(10–76)	CsA/ATG	CsA: 4–6 mg/kg/d,then 150-250/ng/ml; or Horse ATG:10 or 15 mg/kg/d,5 d (Institut Melieux); or 10 or 20mg/kg/d,8 d (Upjohn); or rabbit ATG: 2.5 mg/kg/d, 5 d (Institut Melieux);	4–6	PCR-SSP	–	5	*15:01
**9**	Chen	2007	China	51	30/21	32(12–79)	CsA + ATG	CsA:5mg/kg/d;then 2.5–3mg/kg/d, >3–4 m	6	PCR-SSP	Zhang	6	*15/*15:01
**10**	Mu	2009	China	37	21/16	26 (25–57)	CsA+ATG/ALG	-	4–26	PCR-SSP	Zhang	5	*15/*15:01
**11**	Yang	2004	China	35	-	22(7–55)	CsA+ androgen+TCM	CsA:6mg/kg/d,10 d;then 3mg/kg/d, >3 m	3	PCR-SSP	Zhang	5	*15:01
**12**	Oguz	2002	Turkey	17	–	–	CsA+ ATG +MP	-	–	–	–	4	*15
**13**	Nakao	1994	Japan	59	24/35	56(15–76)	CsA	-	3+	PCR-SSP	–	5	*15:01

AA, aplastic anemia; ALG, antilymphocyte globulin; ATG, antithymocyte globulin; CsA, cyclosporine A; MP, methylprednisolone; PCR-SSO, polymerase chain reaction with sequence-specific oligonucleotide; PCR-SSP, polymerase chain reaction with sequence-specific primer; TCM, traditional Chinese medicine.

### Quantitative synthesis

#### Association of HLA-DRB1*15 and HLA-DRB1*15:*01* polymorphisms with AA

**Fig 2:**
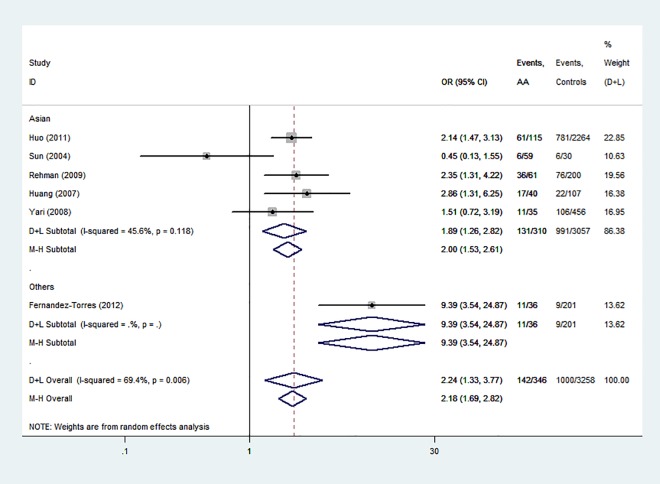
Forest plot of HLA-DRB1*15 polymorphism and aplastic anemia.

**Fig 3:**
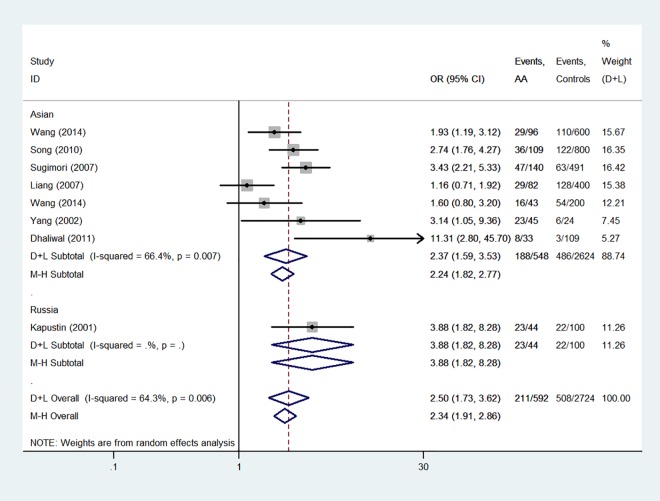
Forest plot of HLA-DRB1*15:*01* polymorphism and aplastic anemia.

The forest plots in Figs 2 and 3 show the main results of the meta-analysis of associations of HLA-DRB1*15 and HLA-DRB1*15:*01* polymorphisms with AA. HLA-DRB1*15 and HLA-DRB1*15:*01* polymorphisms conferred a significantly increased risk. The analysis of the pooled data of six case–control studies revealed a significant increase in the frequency of HLA-DRB1*15 polymorphism (41.0% in AA compared with 30.7% in controls). A moderate level of heterogeneity (*I*^2^ = 69.4%, *P* < 0.01) was found. A random-effects model was used to calculate *OR*. The overall *OR* (95% *CI*) was 2.24(1.33–3.77) with *P* < 0.01. For HLA-DRB1*15:*01* polymorphism (35.6% in AA compared with 18.6% in controls), a moderate level of heterogeneity existed (*I*^2^ = 64.3%, *P* < 0.01). A random-effects model was used. The overall *OR* (95% *CI*) was 2.50(1.73–3.62) with *P* < 0.01.

The subgroup analysis of HLA-DRB1*15 and HLA-DRB1*15:*01* polymorphisms showed similar results. The *OR* (95% *CI*) values of HLA-DRB1*15 and HLA-DRB1*15:*01* were 2.00(1.53–2.61) from fixed—effects model (Fig 2) and 2.37 (1.59–3.53) from random—effects model, respectively, for the Asian patients (*P* < 0.01) (Fig 3).

#### Response to IST in AA

**Fig 4:**
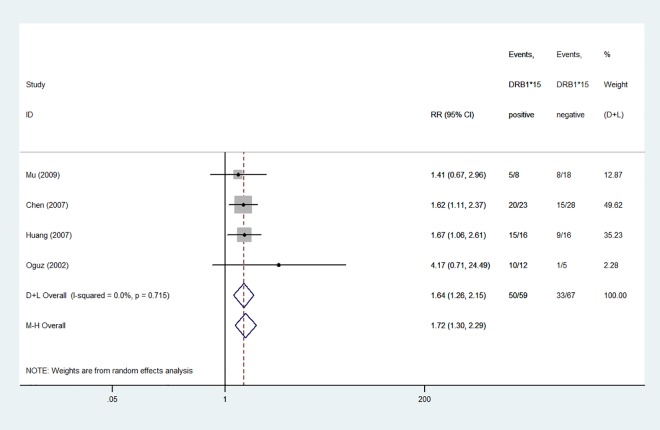
Forest plot of relative ratios for overall response rate between HLA-DRB1*15^*+*^ and HLA-DRB1*15− patients.

**Fig 5:**
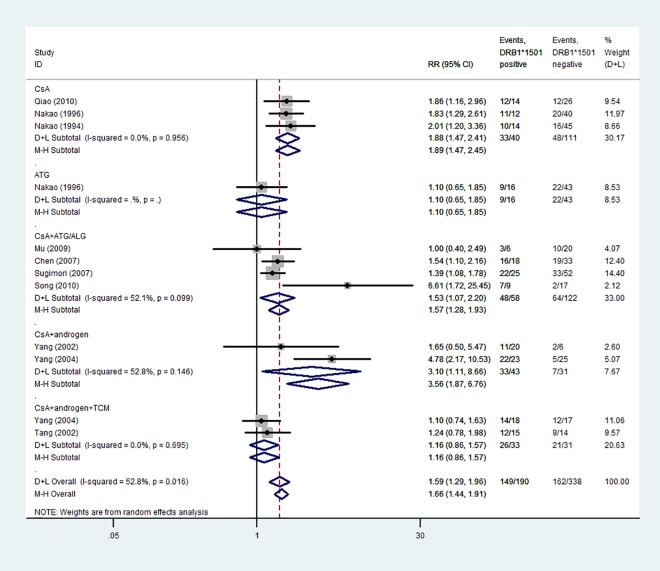
Forest plot of relative ratios for overall response rate between HLA-DRB1*15:*01*^*+*^ and HLA-DRB1*15:*01−* patients.

A summary of the meta-analysis findings on the association of *HLA*-DRB1*15 and *HLA*-DRB1*15:*01* polymorphisms with response to IST in AA is provided in Figs 4 and 5. The response to IST was significantly higher in *HLA*-DRB1*15^*+*^ patients (84.7%) than in *HLA*-DRB1*15− patients (49.3%), with no heterogeneity (*I*^2^ = 0%, *P* = 0.715). A fixed-effects model was used for calculating *RR*. The overall *RR* (95% *CI*) was 1.72 (1.30–2.29) (*P* < 0.01).

For *HLA*-DRB1*15:*01*, the response to IST was significantly higher in *HLA*-DRB1*15:*01*^*+*^ patients (78.4%) than in *HLA*-DRB1*15:*01−* patients (47.9%), with a moderate level of heterogeneity (*I*^2^ = 52.8%, *P* = 0.016). A random-effects model was used for calculating *RR*. The overall *RR* (95% *CI*) was 1.59 (1.29–1.96) (*P* < 0.01).

In the CsA therapy subgroup, an improvement in 33 of 40 *HLA*-DRB1*15:*01*^*+*^ patients was observed, while the response rate was 48/111 in *HLA*-DRB1*15:*01−* patients, with no heterogeneity (*I*^2^ = 0%, *P* = 0.956). A fixed-effects model was used for calculating *RR*. The overall *RR* (95% *CI*) was 1.89 (1.47–2.45) with *P* < 0.01. In the CsA plus ATG/ALG group, a moderate level of heterogeneity was found (*I*^2^ = 52.1%, *P* = 0.099). A random-effects model was used for calculating *RR*. The overall *RR* (95% CI) was 1.53 (1.07–2.20) with *P* < 0.01. In the CsA + androgen group, no heterogeneity was found (*I*^2^ = 52.8%, *P* = 0.146). A fixed-effects model was used for calculating *RR*. The overall *RR* (95% *CI*) was 3.56 (1.87–6.76) with *P* < 0.01. In the CsA + androgen + TCM group, there is no significant difference between groups (Fig 5).

### Sensitivity analyses

A single report involved in the meta-analysis was removed each time to reflect the influence of the individual dataset on the pooled *OR* or *RR*, and the corresponding pooled *OR* and *RR* were not materially changed (data not shown), indicating that the results were statistically robust.

### Publication bias

**Fig 6:**
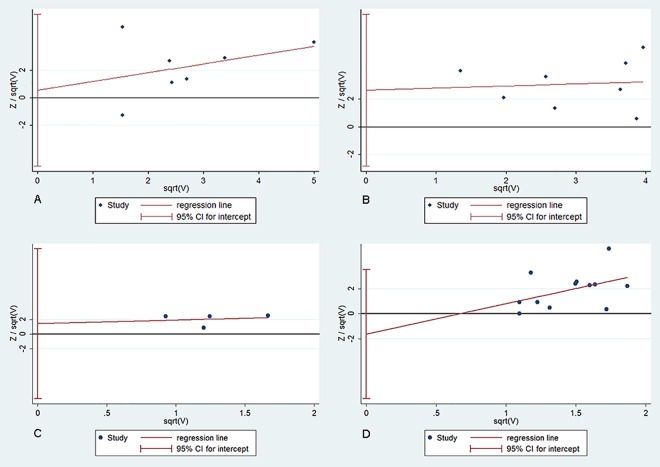
Publication bias plots using the Harbord’s test. (A) Publication bias plot of HLA-DRB1*15 polymorphism and aplastic anemia. (B) Publication bias plot of HLA-DRB1*15:01 polymorphism and aplastic anemia. (C) Publication bias plot of RR between HLA-DRB1*15^*+*^ and HLA-DRB1*15− patients. (D) Publication bias plot of RR between HLA-DRB1*15:*01*^*+*^ and HLA-DRB1*15:*01−* patients.

The shape of the Harbord’s funnel plot showed a relatively symmetric distribution with no publication bias by statistical evidence (*P* > 0.05, shown in Fig 6), indicating that the results of this study were statistically robust.

### Influence of age at diagnosis, region, and NOS score

The results of meta-regression analysis showed that age, region, or NOS score did not account for heterogeneity when studying the association of HLA-DRB1*15 or HLA-DRB1*15:*01* polymorphisms with AA (Table 3).

**Table 3:** Meta-regression.

	HLA-DRB1*15		HLA-DRB1*15:*01*	
	*β*	*P*-value	*β*	*P*-value
Age	-0.093	0.067	-0.001	0.919
Region	1.542	0.092	-0.495	0.484
NOS score	0.098	0.912	-0.784	0.103

## Discussion

This study systematically reviewed the articles on the relationship of *HLA*-DRB1*15 and *HLA*-DRB1*15:*01* polymorphisms with response to IST in AA. Based on the search criteria, 24 studies involving 14 case–control studies and 13 cohort studies were included in the final meta-analysis. A total of 938 cases and 5992 controls from case–control studies were used to find the relationship of *HLA*-DRB1*15 and *HLA*-DRB1*15:*01* polymorphisms with AA in the pooled analyses. Moreover, 609 AA in cohort studies were used to discuss the association of *HLA*-DRB1*15 and *HLA*-DRB1*15:*01* polymorphisms with response to IST in AA. This study was perhaps the first meta-analysis to explore the relationship of DRB1*15 and DRB1*15:*01* with response to IST in AA.

Of the six studies about the associations between *HLA*-DRB1*15 polymorphisms and AA and eight studies about the associations between *HLA*-DRB1*15:*01* polymorphisms and AA, the majority indicated that *HLA*-DRB1*15 and *HLA*-DRB1*15:*01* polymorphisms might be potential risk factors for AA [6–10, 14–15, 18–19, 24], but four studies indicated no association of *HLA*-DRB1*15 and *HLA*-DRB1*15:01 polymorphisms with AA [5–6, 12–13,16]. The pooled results of the meta-analysis were consistent with most studies, which indicated *HLA*-DRB1*15 and *HLA*-DRB1*15:01 polymorphisms as potential risk factors for AA (*OR* = 2.24, 2.50, respectively; Figs 2 and 3). In subgroup analysis, region was the reason for heterogeneity in *HLA*-DRB1*15 but not in *HLA*-DRB1*15:*01*. In addtion, there was only one small non-Asians group in each analysis. So it is more valid to say that these associations were found in Asian populations.

Of the 4 studies about the associations between *HLA*-DRB1*15 polymorphisms and response to IST in AA and 11 studies about the associations between *HLA*-DRB1*15:*01* polymorphisms and response to IST in AA, the majority indicated that patients with AA who carried *HLA*-DRB1*15 or *HLA*-DRB1*15:*01 alleles* might have a good response rate for the IST [14–15, 23–24, 26–27, 30–31]. Seven studies indicated no association of *HLA*-DRB1*15 and *HLA*-DRB1*15:*01* polymorphisms with IST in AA [17–18, 25, 28–30]. The pooled results showed that the response rate was significantly higher in *HLA*-DRB1*15^*+*^ and *HLA*-DRB1*15:*01*^*+*^ patients than in *HLA*-DRB1*15− and *HLA*-DRB1*15:*01−* patients (84.7% vs 49.3% and 78.4% vs 47.9%, *RR* = 1.72 and 1.59, respectively). It means that *HLA*-DRB1*15^*+*^ and *HLA*-DRB1*15:*01*^*+*^ patients with AA treated with IST were more sensitive than *HLA*-DRB1*15− and *HLA*-DRB1*15:*01−* patients.

In a subgroup analysis, *HLA*-DRB1*15:*01*^*+*^ patients treated with CsA, CsA + ATG/ALG, or CsA + androgen showed a higher response rate than DRB1*15:*01−* patients (*RR* = 1.89, 1.53 and 3.56, respectively). Only two articles included the CsA + androgen subgroup. Hence, the results needed further investigation. A negative result was obtained in CsA + androgen + TCM group. It is known that TCM combine different kinds of herbs that might have cause the clinical heterogeneity. Further researches and analyses are needed to validate the findings.

Heterogeneity is a potential issue that may affect the results of all meta-analyses. Statistical heterogeneity existed among some analyses in the present study. Several methods were applied to examine whether the results were robust. First, we considered region, mean or median age or NOS score as a covariate in the meta-regression analysis. The results indicated that these factors are not statistically significant (P>0.05) for heterogeneity when studying the association between DRB1*15 or DRB1*15:*01* polymorphisms and AA. Second, subgroup analyses by region or drugs and sensitivity analyses were performed. It indicated that drug groups led to heterogeneity when studying the association of *HLA*-DRB1*1501 polymorphisms with response to IST in patients with AA. Additionally, the region was a significant factor for heterogeneity when studying the association between *HLA*-DRB1*15 polymorphisms and AA. However, the region was not a significant factor for heterogeneity when studying the association between *HLA*-DRB1*15:*01* polymorphisms and AA.

Both English and Chinese language reports were identified, obtained, and included in this analysis to avoid the local literature bias[40]. However, several limitations still could not be ignored. First, the results were based on unadjusted analysis. Some factors such as the dose, product and biological characteristics of the xenoantisera, short telomeres, younger age, absolute reticulocyte count, absolute lymphocyte count, normal cytogenetics, and paroxysmal nocturnal hemoglobinuria clone, were also associated with a higher response rate [1, 41]. However, information was not available to perform more detailed analysis. As a result, these factors were not considered in this study. Further researches are still needed in the future to figure out the complex effect of the aforementioned factors and *HLA*-DRB1*15, *and HLA*-DRB1*15:*01* polymorphisms. Second, HLA typing was performed by PCR with sequence-specific primers in most included articles, but two reports involved PCR with sequence-specific oligonucleotide primers. The typing methods were not identical between different researches, which might have led to the heterogeneity in the present analysis. Third, probably most of the *HLA*-DRB1*15 patients were actually *HLA*-DRB1*15:*01*, but it was not confirmed. Finally, because of the low incidence of *DRB1* genotype, limited studies were available for inclusion in this meta-analysis. Only few articles were found about other ethnicities. Hence, it could not be concluded whether *HLA*-DRB1*15 and *HLA*-DRB1*15:*01* polymorphisms were different in those ethnic groups.

## Conclusions

*HLA*-DRB1*15 and *HLA*-DRB1*15:*01* polymorphisms might be associated with increased AA risk in Asians. IST might be more effective in Asian patients with *HLA*-DRB1*15 and *HLA*-DRB1*15:*01* polymorphisms than in Asian patients without *HLA*-DRB1*15 and *HLA*-DRB1*15:*01* polymorphisms. More articles with adequate methodological quality on gene–gene and gene–environment interactions and gene treatment may eventually lead to valid results in the future.

## Supporting Information

S1 File Protocol of the research. (PDF)

S2 File Search strategy. (DOCX)

S3 File Certificate of English editing. (JPG)

S1 Table PRISMA Checklist. (DOC)

S2 Table Genetic association meta-analysis checklist. (DOC)

S3 Table Reasons for exclusion (DOC)
