# Supplementary material for: Association of Human Leukocyte Antigen DRB1*15 and DRB1*15:01 Polymorphisms with Response to Immunosuppressive Therapy in Patients with Aplastic Anemia: A Meta-Analysis
Source: PLoS One. 2016 Sep 9;11(9):e0162382. doi: 10.1371/journal.pone.0162382 (PMC5017877; doi:10.1371/journal.pone.0162382)
Supplement: S1 File — (PDF) [file pone.0162382.s001.pdf]

PROSPERO International prospective register of systematic reviews

Association of human leukocyte antigen DRB1*15/1501 with response to immunosuppressive therapy in aplastic anemia patients: a meta-analysis

Shan Liu, Conghua Ji

Citation

Shan Liu, Conghua Ji. Association of human leukocyte antigen DRB1*15/1501 with response to immunosuppressive therapy in aplastic anemia patients: a meta-analysis. PROSPERO 2015:CRD42015032293 Available from http://www.crd.york.ac.uk/PROSPERO_REBRANDING/display_record.asp?ID=CRD42015032293

Review question(s)

We aimed to systematically evaluate the association of HLA-DRB1*15/1501 with response to immunosuppressive therapy in aplastic anemia patients.

Searches

Several databases (MEDLINE/PubMed, China National Knowledge Infrastructure CNKI, EMBASE, Web of Science and Cochrane databases) will be searched through October 2015 for all publications on the association between HLA polymorphism and immunosuppressive therapy in AA.

The search terms will be as follows: (“Aplastic anemia”) and(“HLA” or “human leukocyte antigen”) and(“polymorphism” or “variant” or “genotype”) and ("cyclosporine" or "antilymphocyte serum" or "immunosuppression" or "anti-rejection therapy" or "antithymocyte globulin" or "antilymphocyte globulin").

No language limitations will be used.

In addition, we will also search references of retrieved reports and contact study authors by e-mail to identify additional studies and ask for missing data.

Types of study to be included

Cohort studies relating to the association of HLA-DRB1*15/1501 polymorphism and the response to immunosuppressive therapy in aplastic anemia patients.

Condition or domain being studied

Papers involving study of HLA-DRB1*15/1501 expression in AA and response to immunosuppressive therapy.

Participants/ population

AA patients with response to immunosuppressive therapy regardless of sex, age and ethnicity will be included.

Intervention(s), exposure(s)

AA patients typed for HLA-DRB1*15/1501 positive comprised the exposures group.

Comparator(s)/ control

AA patients typed for HLA-DRB1*15/1501 negative comprised the non-exposures group.

Outcome(s)

Primary outcomes

Response rate at six months from the start of treatment.

Secondary outcomes

None.

Risk of bias (quality) assessment

Quality of the included articles will be evaluated using the Newcastle-Ottawa Scale (NOS) scale. The NOS contains eight items categorized into three dimensions including selection, comparability, and exposure. For each item a series of response options is provided. A star system is used to allow a semi-quantitative assessment of study quality, such that the highest quality studies are awarded a maximum of one star for each item with the exception of the item related to comparability that allows the assignment of two stars. The NOS ranges between zero and nine stars.

Strategy for data synthesis

Review manager 5.3 software will be used to perform the meta-analysis. Heterogeneity will be checked by the Chi-square test (Cochran 1954) and the I-squared statistic (Higgins 2003). The criteria for identification of heterogeneity is a P value less than 0.10 for the Chi-square test and an I-squared statistic greater than 50%. When there is no statistical evidence for heterogeneity in effect sizes, we will use the fixed-effect model (Mantel 1959). When significant heterogeneity is identified, we will use the random-effects model (DerSimonian 1986) and explore sources of significant heterogeneity.

Analysis of subgroups or subsets

Subgroup analyses will be performed by ethnicity.

Contact details for further information

Shan Liu

Room 319

Post Road 23

Shangcheng District

Hangzhou City

China

graystar92@163.com

Organisational affiliation of the review

None

Review team

Dr Shan Liu,
Professor Conghua Ji,

Anticipated or actual start date

04 October 2015

Anticipated completion date

29 February 2016

Funding sources/sponsors

None

Conflicts of interest

None known

Language

English

Country
China

Subject index terms status
Subject indexing assigned by CRD

Subject index terms
Anemia, Aplastic; Histocompatibility Antigens Class II; Histocompatibility Testing; HLA Antigens; Humans; Immunosuppressive Agents; Treatment Outcome

Stage of review
Ongoing

Date of registration in PROSPERO
15 December 2015

Date of publication of this revision
15 December 2015

**Stage of review at time of this submission**	**Started**	**Completed**
Preliminary searches	Yes	Yes
Piloting of the study selection process	Yes	No
Formal screening of search results against eligibility criteria	Yes	No
Data extraction	No	No
Risk of bias (quality) assessment	No	No
Data analysis	No	No

PROSPERO
International prospective register of systematic reviews
The information in this record has been provided by the named contact for this review. CRD has accepted this information in good faith and registered the review in PROSPERO. CRD bears no responsibility or liability for the content of this registration record, any associated files or external websites.
