# Supplementary material for: Association of Human Leukocyte Antigen DRB1*15 and DRB1*15:01 Polymorphisms with Response to Immunosuppressive Therapy in Patients with Aplastic Anemia: A Meta-Analysis
Source: PLoS One. 2016 Sep 9;11(9):e0162382. doi: 10.1371/journal.pone.0162382 (PMC5017877; doi:10.1371/journal.pone.0162382)
Supplement: S2 File — (DOCX) [file pone.0162382.s002.docx]

| Database | Strategy | Number |
| --- | --- | --- |
| CNKI | SU=('再生障碍性贫血'+'再障') and SU=('HLA'+'人白细胞抗原'+'人类白细胞抗原') and SU=('基因'+'多态性'+'变异') | 215 |
| Wangfang | KeyWords=(再生障碍性贫血 or 再障) and KeyWords=(HLA or 人白细胞抗原 or 人类白细胞抗原) | 29 |
| VIP | (Keyword_C=再生障碍性贫血+再障) 与 (Keyword_C= HLA+人白细胞抗原+人类白细胞抗原) | 74 |
| CBM | 缺省=(再生障碍性贫血 or 再障) and 缺省=(HLA or 人白细胞抗原 or 人类白细胞抗原) | 234 |
| Pubmed | Search (((((("Cyclosporine"[Mesh]) OR "Antilymphocyte Serum"[Mesh]) OR "Immunosuppression"[Mesh]) OR (((((((Immunosuppressions[Title/Abstract]) OR Anti-Rejection Therapy[Title/Abstract]) OR Antirejection Therapy[Title/Abstract]) OR Cyclosporine[Title/Abstract]) OR Antilymphocyte Serum[Title/Abstract]) OR antithymocyte globulin[Title/Abstract]) OR antilymphocyte globulin[Title/Abstract]))) AND (((human leukocyte antigen[Title/Abstract]) OR "HLA Antigens"[Mesh]) OR "Major Histocompatibility Complex"[Mesh])) AND ((aplastic anemia[Title/Abstract]) OR "Anemia, Aplastic"[Mesh]) | 738 |
| Cochrane Library | #1 MeSH descriptor: [HLA Antigens] explode all trees  #2 MeSH descriptor: [Major Histocompatibility Complex] explode all trees  #3 human leukocyte antigen  #4 #1 or #2 or #3  #5 aplastic anemia  #6 MeSH descriptor: [Anemia, Aplastic] explode all trees  #7 #5 or #6  #8 #4 AND #7  #9 MeSH descriptor: [Cyclosporine] explode all trees  #10 MeSH descriptor: [Antilymphocyte Serum] explode all trees  #11 MeSH descriptor: [Immunosuppression] explode all trees  #12 Immunosuppressions  #13 Anti-Rejection Therapy  #14 Antirejection Therapy  #15 Cyclosporine  #16 Antilymphocyte Serum  #17 antithymocyte globulin  #18 antilymphocyte globulin  #19 #9 or #10 or #11 or #12 or #13 or #14 or #15 or #16 or #17 or #18  #20 #4 and #7 and #19  #21 #8 or #20 | 22 |
| EMBASE | #1 exp HLA antigen/ or human leukocyte antigen.mp. or exp HLA system/  #2 Major Histocompatibility Complex.mp. or exp major histocompatibility complex/  #3 #1 or #2  #4 aplastic anemia.mp. or exp aplastic anemia/  #5 exp HLA DR antigen/ or exp HLA DRB1 antigen/ or drb1.mp.  #6 #3 and #4 and #5  #7 Cyclosporine.mp. or exp cyclosporin/  #8 Immunosuppression.mp. or immunosuppressive treatment/  #9 immunosuppressive treatment/ or Anti-Rejection Therapy.mp. or exp cyclosporin A/  #10 Antilymphocyte Serum.mp. or exp lymphocyte antibody/  #11 antithymocyte globulin.mp. or exp thymocyte antibody/  #12 antilymphocyte globulin.mp. or exp lymphocyte antibody/  #13 #7 or #8 or #9 or #10 or #11 or #12  #14 #6 and #13  #15 #6 or #14 | 262 |
