# Supplementary figures and images for: Association of Human Leukocyte Antigen DRB1*15 and DRB1*15:01 Polymorphisms with Response to Immunosuppressive Therapy in Patients with Aplastic Anemia: A Meta-Analysis
Source: PLoS One. 2016 Sep 9;11(9):e0162382. doi: 10.1371/journal.pone.0162382 (PMC5017877; doi:10.1371/journal.pone.0162382)

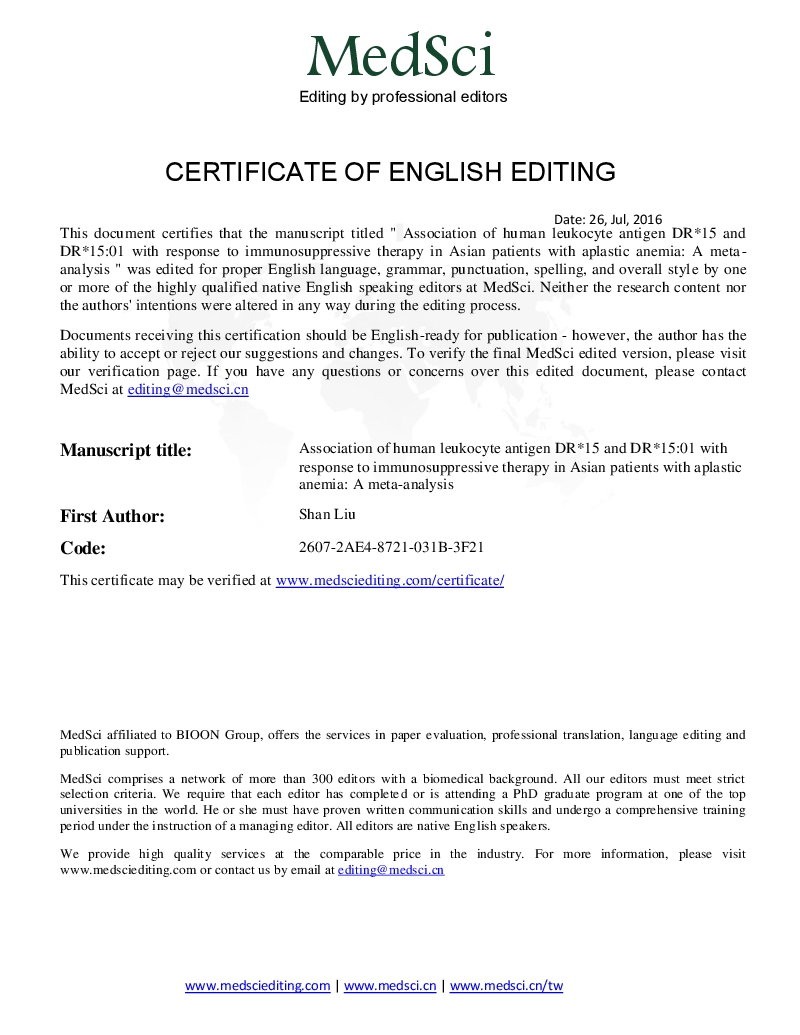

Supplement: S3 File — (JPG) [file pone.0162382.s003.jpg]
