# Supplementary material for: Association of Human Leukocyte Antigen DRB1*15 and DRB1*15:01 Polymorphisms with Response to Immunosuppressive Therapy in Patients with Aplastic Anemia: A Meta-Analysis
Source: PLoS One. 2016 Sep 9;11(9):e0162382. doi: 10.1371/journal.pone.0162382 (PMC5017877; doi:10.1371/journal.pone.0162382)
Supplement: S3 Table — (DOC) [file pone.0162382.s006.doc]

Supplementary Table. Reasons for exclusion.

| **No** | **Study(Author, year)** | **Reason(s) for exclusion** |
| --- | --- | --- |
| 1 | Itoh 2005 | HLA typing was insufficient and study design was not case-control |
| 2 | Nimer 1994 | HLA typing was insufficient |
| 3 | Levy 2010 | Genome-wide association analysis |
| 4 | Laundy 2004 | Study on prevalence and characteristics of anti-HLA in antibody |
| 5 | Battiwalla 2012 | HLA typing was insufficient and study design was not case-control |
| 6 | Shao 2000 | Insufficient genetic data |
| 7 | Peinemann 2014 | HLA typing was insufficient and study design was not case-control |
| 8 | Ding 2011 | Review |
| 9 | Shao 1998 | Review |
| 10 | Huang 2006 | HLA typing was insufficient and study design was not case-control |
| 11 | Tang 2014 | HLA typing was insufficient and study design was not case-control |
| 12 | Yu 2006 | HLA typing was insufficient |
| 13 | Song 1995 | Study on expression of HLA-DR [antigen](javascript:void(0);) |
| 14 | Yang 1997 | Only HLA-A,-B, and-DQ alleles were typed |
| 15 | Yang 1997 | Only HLA-A,-B, and-DQ alleles were typed |
| 16 | Chen 2007 | HLA typing was insufficient and study design was not case-control |
| 17 | Chen 2005 | HLA typing was insufficient and study design was not cohort |
| 18 | Song 1997 | Duplicate publication |
| 19 | Qiao 2010 | Duplicate publication |
| 20 | Chen 2007 | Duplicate publication |
| 21 | Lu 2012 | Duplicate publication |
| 22 | Katagiri 2012 | Written in Japanese |
| 23 | Zhang 2012 | HLA typing was insufficient and study design was not cohort |
| 24 | Sun 2002 | Review |
| 25 | Shichishima 2015 | Aplastic anemia-paroxysmal nocturnal hemoglobinuria syndrome |
| 26 | Sauthararajah 2015 | HLA typing was insufficient |
| 27 | Ihan 1997 | HLA typing was insufficient |
| 28 | Usman 2004 | HLA typing was insufficient |
| 29 | Chen 2012 | Insufficient genetic data |
| 30 | Lu 2012 | Insufficient genetic data |
| 31 | Nowak2010 | HLA haplotypes |
| 32 | Lee 1990 | Review |
| 33 | Nakao 1994 | HLA haplotypes |
| 34 | Rugman 1990 | HLA typing was insufficient |
| 35 | Sun 2000 | Insufficient genetic data |
| 36 | Sun 2002 | Insufficient genetic data |
| 37 | Shao 1999 | HLA typing was insufficient |
| 38 | Katagiri 2011 | HLA typing was insufficient |
| 39 | Kim 1986 | HLA haplotypes |
| 40 | Yang 2003 | Duplicate publication |
| 41 | Sun 2005 | Duplicate publication |
| 42 | Yang 2002 | Duplicate publication |
| 43 | Shao 2000 | Duplicate publication |
| 44 | Schrezenmeier 2015 | Review |
